# “And then I found $5”: Optimizing recruitment efficiency in remote clinical trials

**DOI:** 10.1017/cts.2023.533

**Published:** 2023-04-20

**Authors:** Margaret C. Fahey, Jennifer Dahne, Brian K. Chen, Tracy T. Smith, Amy E. Wahlquist, Mathew J. Carpenter

**Affiliations:** 1 Department of Psychiatry & Behavioral Sciences, Medical University of South Carolina, Charleston, SC, USA; 2 Hollings Cancer Center, Medical University of South Carolina, Charleston, SC, USA; 3 Arnold School of Public Health, University of South Carolina, Columbia, SC, USA; 4 Center for Rural Health Research, East Tennessee State University, Johnson City, TN, USA

**Keywords:** Remote trials, technology-based informed consent, e-consent, mail-based informed consent, enrollment procedures

## Abstract

**Introduction:**

As clinical trials adopt remote methodologies, there is need to optimize efficiency of remote enrollment. Within a remote clinical trial, we aim to (1) assess if sociodemographic factors differ among those consenting via mail vs. technology-based procedures (e-consent), (2) determine if, among those consenting via mail, a small unconditional monetary reward ($5) increases likelihood of subsequent enrollment, (3) economically evaluate additional cost per additional participant enrolled with $5 reward.

**Methods:**

In the parent nationwide randomized clinical trial of adult smokers (*N* = 638), participants could enroll via mail or e-consent. Logistic regression models assessed relationships between sociodemographics and enrollment via mail (vs e-consent). Mailed consent packets were randomized (1:4) to include $5 unconditional reward or not, and logistic regression modeling examined impact of reward on subsequent enrollment, allowing for a randomized study within a study. Incremental cost-effectiveness ratio analysis estimated additional cost per additional participant enrolled with $5 incentive.

**Results:**

Older age, less education, lower income, and female sex predicted enrolling via mail vs e-consent (*p* < .05’s). In adjusted model, older age (AOR = 1.02, *p* = .016) and less education (AOR = 2.23, *p* < .001) remained predictive of mail enrollment. The $5 incentive (vs none) increased enrollment rate by 9% (AOR = 1.64, *p* = .007), with estimated cost of additional $59 per additional participant enrolled.

**Conclusions:**

As e-consent methods become more common, they have potential to reach many individuals but with perhaps diminished inclusion across all sociodemographic groups. Provision of an unconditional monetary incentive is possibly a cost-effective mechanism to increase recruitment efficiency for studies employing mail-based consenting procedures.

## Introduction

One goal of clinical trials is to recruit a sufficiently large and representative study sample, allowing for valid and generalizable results. Moreover, clinical trials need efficient recruitment procedures to obtain the targeted sample size within budget and time constraints. However, recruitment is a common challenge for many trials. Two reviews suggest only slightly more than half (55%–56%) of all randomized controlled trials, across a range of health-related topics, reach their targeted sample size [1,2]. Under-recruitment has the potential for false-negative results, insofar that these trials are insufficiently powered to detect intended treatment effects.

Remote clinical trials can improve recruitment efficiency by expanding geographic study reach and reducing barriers of participation [3,4]. With advancements in technology, remote trials significantly reduce or even remove logistical and time constraints of in-person procedures. All trials, whether remote or in-person, formally begin through a process to obtain informed consent. In the case of remote trials, this is often managed through electronic consent (e-consent) [5]. E-consent enables participants to sign and date consent via a variety of electronic interfaces (e.g., websites, tablets, mobile devices) [5]. E-consent can be conducted in-person or within teleconsent, in which participants “meet” with research staff remotely (e.g., phone, virtual platform) for an interactive process that addresses any participant questions [5]. Yet, some participants lack access and skills for using the technology required for e-consent procedures. Certain demographic characteristics (older age, rurality, lower income, less education) are associated with lower rates of mobile device ownership and access to home broadband Internet access, both of which can undermine capacity for e-consent completion [6–8]. Further, some individuals might prefer to sign consent forms on paper, rather than use technology [9,10]. Thus, while e-consents offer significant opportunity to expand study access, there are barriers to this type of enrollment.

Prior to technological advancements, remote trials relied largely on mail-based procedures for obtaining informed consent. Given back-and-forth mailing, this approach often extends the recruitment process. In addition, mail-based consenting is susceptible to error (e.g., incomplete/unsigned forms, erroneous date of signing) and non-response. For example, in our prior remote trial, half of all mailed consent packets were unreturned [11]. To enhance efficiency of mailed consent procedures, one option is to offer a prepaid unconditional (i.e., not dependent on any behavior) monetary reward within the mailed packet. This is a well-known practice within telephone marketing surveys, where an unconditional prepaid incentive ($1–$20 cash) can increase response rate by 10% [12]. However, less is known about the impact of an unconditional monetary incentive on participant recruitment within clinical trials.

A 2018 review of recruitment strategies in randomized clinical trials (RCTs) identified two published studies that assessed the impact of a financial incentive on enrollment [13], suggesting an increase in enrollment of 4% [13]. Yet, this review deemed the evidence moderate given the few studies on this topic and inconsistency between study methodologies (i.e., unconditional €5 vs conditional €100 upon consenting). Since the prior review, only one study, to our knowledge, randomized potential participants to receive an unconditional monetary incentive (€5) vs. no incentive in the context of a larger ongoing clinical trial [14], yielding a small impact on enrollment (3.4% with incentive vs 2.9%). Given the lack of research in this area and inconsistency of findings, more research is needed to assess the impact of unconditional monetary incentives on study enrollment in a clinical trial.

The current study was conducted within a nationwide remote RCT among adults who smoke cigarettes. The parent trial recruited participants through either mail-based or e-consent processes (non-randomized, based on preference and technological capacity). Among those enrolling via mail, potential enrollees were randomized to receive a small unconditional monetary incentive ($5 cash) or no incentive. The current paper had three aims: (1) to compare sociodemographic characteristics of those who enrolled via mail vs e-consent and (2) assess if the monetary reward ($5) increased likelihood of study enrollment among mail enrollees. To inform budgetary considerations of future trials, our final aim (3) was to economically evaluate the additional cost per additional participant enrolled with the implementation of the $5 unconditional reward.

## Materials & Methods

### Eligibility and Recruitment

The parent study was a nationwide RCT that examined the naturalistic impact of e-cigarette use among adults who smoked cigarettes (*N* = 638). Recruitment (2018–2022) occurred online through social media advertising within cities selected specifically for high racial, ethnic, and geographic diversity. Each city had a limit of 80 participants, with a quota of at least 30% non-White participants, to further ensure demographic diversity. Eligibility criteria included ≥ 21 years of age, currently smoking ≥ 5 cigarettes per day for ≥ one year, not pregnant, no recent cardiovascular distress or history of renal disease or seizure disorder, at least some concern about the health effects of cigarette smoking, not currently using cigarette cessation medication, had not purchased or regularly used (daily or weekly) an electronic nicotine delivery system product in the past six months, no current use of other (non-cigarette) tobacco products, and regular use of email and/or a mobile device with the capacity to receive SMS text and Internet access. Those interested were directed to an online secure screening form to assess eligibility.

### Procedures

**Screening.** Online screening determined study eligibility and interest. At screening, potential participants reported their preferred method and/or technological capacity (i.e., webcam access and/or compatible Internet browser) for informed consent via either (1) mail, or (2) technology-based e-consent. All consenting procedures were approved by local IRB review.

**E-consent.** E-consent included either (a) real-time, personalized online consent through doxy.me or (b) e-consent via REDCap, both requiring Internet access [15,16]. Doxy.me is a video-based platform that requires both webcam and speakers. REDCap consent did not involve any video engagement with the participant and relied solely on real-time telephone contact between research staff and participant. Both options allowed participants to digitally sign and date the consent form on their computer, which could be synchronously facilitated and seen by research staff. Upon completion, e-consents were stored electronically within secure, password-protected files.

**Mail-based consent**. Those opting to provide consent via mail and/or who did not have technological capacity for e-consent received a consent packet in the mail. This packet contained two copies of the consent form, a baseline questionnaire, a pre-addressed and pre-stamped return envelope, and information for a toll-free phone line to ask any study-related questions. Mailed consent packets included instructions to sign and return one consent form and the completed baseline questionnaire. Consistent with IRB approvals at the time of this study, mail-based enrollment did not require a synchronous phone call with study staff. This was dissimilar from e-consent, in which a synchronous discussion with study staff was required when electronically signing consent.

**Unconditional monetary incentive**. To assess the recruitment impact from an unconditional reward, a subset of mailed consent packets included $5 cash, not linked to any behavior. Randomization to monetary incentive was 1:4, such that 20% of potential participants received reward, and 80% did not. A 1:4 randomization was chosen for budget considerations.

### Measures

**Sociodemographic characteristics.** During online eligibility screening (prior to informed consent), potential participants reported standard demographic characteristics to guide CONSORT reporting including age, sex, race, and ethnicity. Following consent, participants completed a baseline questionnaire, in which they again reported age, sex, race, and ethnicity, as well as additional demographics: marital status, educational background, income, and employment status. Based on participant zip codes, we determined the primary Rural-Urban Commuting Area (RUCA) codes (2010) for each participant [17]. Primary RUCA codes measure rurality based on the size and direction of primary commuting patterns. RUCA codes range from 1 (metropolitan) to 10 (rural). RUCA scores were dichotomized as 1–3 (metropolitan) and 4–10 (micropolitan, small town, rural) [17].

**Consent type.** We defined e-consent enrollment as consenting using a technology-based procedure [either via doxy.me (18.8% of all enrollees, *n* = 120) or REDCap (42.6% of all enrollees, *n* = 272)] and subsequently enrolling into the study by completing baseline visit. We combined doxy.me and REDCap consenting groups because the focus of this study was to compare those using mail-based vs. technology-based consent procedures, rather than to compare different modalities of e-consent. No sociodemographic differences were found between those opting for doxy.me vs. REDCap procedures. We defined mail-based enrollment as receiving consent packets in the mail (i.e., excluding those with returned/undeliverable addresses), returning the packet in full, and subsequent enrollment via completion of baseline visit. The decision for mail vs e-consent was based on (a) brief assessment of capacity (i.e., webcam access and/or compatible Internet browser for REDCap) and, if met, (b) participant preference. Consent type is relevant for Analysis 1.

**Recruitment costs**. We determined total recruitment costs by adding the cost of advertising and the labor cost of recruitment personnel.

## Data Analyses

### Analysis 1: Sociodemographic Characteristics of Mail-Based Enrollment (vs E-Consent)

For the first aim, among those who enrolled in the study (*N* = 638), unadjusted binary logistic regression models examined the association between sociodemographic characteristics and the likelihood of enrolling in the trial via mail (vs e-consent). Sociodemographic characteristics were coded as age (continuous), marital status [married/partnered or not married (divorced, separated, widowed, single and never married)], race (White, non-White), ethnicity (Hispanic/Latino, non-Hispanic/Latino), income (<$50,000, ≥ $50,000), educational background (some college or less, college degree or more), sex (male, female), employment status (unemployed/retired, employed full or part time), and RUCA code (metropolitan vs. micropolitan/small town/rural). A final adjusted binary logistic regression model included all significant sociodemographic characteristics from the unadjusted analyses (*p* < .05) to predict enrolling via mail (vs e-consent).

### Analysis 2: Impact of $5 Unconditional Incentive

We provide descriptive statistics on demographic characteristics (based on eligibility screening) for all participants who preferred consent via mail and/or who did not have technological capacity for e-consent and subsequently received a consent packet in the mail. (*N* = 948). This sample size is larger than the enrolled sample above (*N* = 638) because more individuals were initially mailed consent forms than enrolled, in anticipation of non-response. Sociodemographic characteristics for this analysis are based solely on information captured during online screening prior to consent (since not everyone subsequently enrolled) and thus are limited to include age (continuous), sex (female, male), race (White, non-White), and ethnicity (Hispanic/Latino, non-Hispanic/Latino). Logistic regression models examined whether those who received the unconditional $5 incentive had a higher likelihood of subsequent enrollment, adjusting for any significant sociodemographic covariates of enrollment (*p* < .05).

### Analysis 3: Incremental Cost-Effectiveness Analysis

To quantify the benefit (i.e., value) of the cash incentive, we calculated an incremental cost-effectiveness ratio (ICER) in terms of the additional cost per additional participant enrolled when a $5 cash incentive was implemented vs. no incentive. In our parent trial, we had unequal numbers of participants in the incentive vs. non-incentive groups (*n* = 138 with incentive vs. *n* = 765 with no incentive). Therefore, we normalized the two groups to 1000 participants each. We calculated the *difference in number of enrolled participants* between the two groups by applying the observed proportions enrolled (32.8% for the incentive group and 24.3% for the non-incentive group) to these normalized participant numbers and taking the difference. (Specifically, 1000 * 32.8%–1000 * 24.3% = 85 additional participants in the $5 incentive group). To calculate the *difference in intervention costs*, we again normalized the groups so that the $5 incentive group with 1,000 participants cost $5000 more than the non-incentive group. We ignored costs common to both groups, such as recruitment costs, as they would be differenced out when we conduct an incremental analysis. We did not vary the incentive amount in this study and so cannot predict the potential effect of incentives of different dollar amounts. Thus, for these ICER calculations, the difference in implementation cost between the incentive and non-incentive groups was fixed at $5 per person without variation. To calculate the ICER, we divided the difference in intervention costs by the difference in number of participants enrolled. Finally, we provided a 95% “confidence interval” around our ICER by using a chi-square difference in proportion test to establish the lower and upper bounds of differences in the number of participants enrolled. We calculated the ICERs at both the lower and upper bounds of these differences.

## Results

### Analysis 1: Sociodemographic Comparisons of Enrollment Via Mailed Consent (vs E-Consent)

**Sample.** Table 1 provides descriptive statistics of sociodemographics for those who enrolled via mailed consent (38.6*%*; *N* = 246) and those who enrolled via e-consent (61.4*%*; *N* = 392).

**Table 1.** Demographic characteristics of study samples

Sociodemographic characteristics	Analysis 1: Enrolled (*N* = 638)		Analysis 2: Received mailed consent packets (*N* = 948)
	Enrolled via mail-based consent (*N* = 246)	Enrolled via E-Consent (*N* = 392)	
Age* *M (SD*)	43.5 (11.3)	41.5 (11.5)	40.1 (10.9)
Sex* *N (%)*			
Male	101 (41.1)	195 (49.7)	442 (46.6)
Female	145 (58.9)	197 (50.3)	506 (53.4)
Race* *N (%)*			
White	171 (69.5)	266 (67.9)	650 (68.6)
non-White	75 (30.5)	126 (32.1)	298 (31.4)
Ethnicity* *N (%)*			
Non-Hispanic/Latino	204 (82.9)	344 (87.8)	788 (83.1)
Hispanic/Latino	42 (17.1)	48 (12.2)	160 (16.9)
RUCA**			
Metropolitan	236 (95.9)	376 (95.9)	–
Micropolitan/small town/rural	10 (4.1)	16 (4.1)	–
Income** *N (%)*			
Less than $50,000	170 (74.2)	245 (64.5)	–
$50,000 or more	59 (25.8)	135 (35.5)	–
Education** *N (%)*			
Some college or less	217 (88.2)	295 (75.3)	–
College degree or more	29 (11.8)	97 (24.7)	–
Marital Status** *N (%)*			
Not married	181 (73.6)	267 (68.1)	–
Married	65 (26.4)	125 (31.9)	–
Employment Status** *N (%)*			
Not employed	112 (45.9)	161 (41.1)	–
Employed	132 (54.1)	231 (58.9)	–

* Only age, sex, race, and ethnicity were assessed at screening, (*N* = 948).

** Remaining sociodemographic characteristics were captured post-consent, within baseline visit. RUCA = Rural-Urban Commuting Area Code (Range: 1-10); Metropolitan = 1-3; Micropolitan/small town/rural = 4-10.

**Figure 1.**
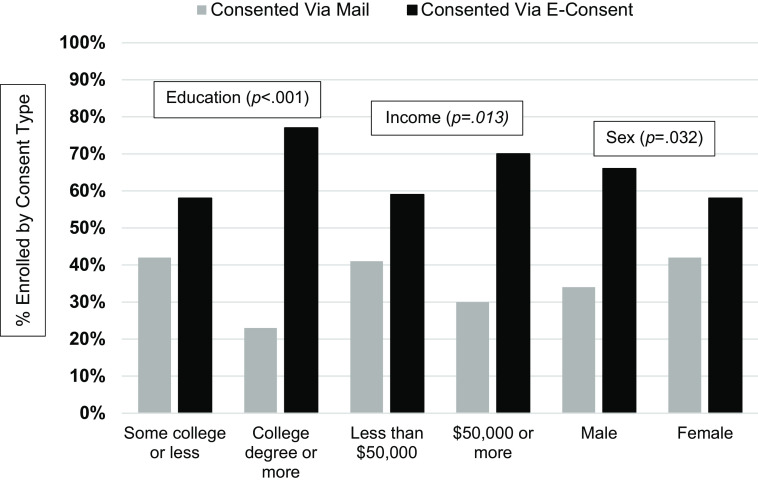
Analysis 1: Enrollment type by categorical sociodemographic characteristics. *Note*: *p*-values represent significance of unadjusted models. Additionally (not shown in graph), increasing age (measured continuously) significantly predicted enrolling via mailed consent vs. e-consent (OR = 1.02, *p* = .035). Rurality, race, ethnicity, marital status, and employment status were not related to enrollment via mail (vs e-consent) (*p* > .05’s).

**Unadjusted Models.** Older age (*p* = .035), less education (*p* < .001), lower income (*p* = .013), and female sex (*p* = .032) each predicted likelihood of enrolling via mail (vs. e-consent) in unadjusted univariate models (see Table 2; Fig. 1).

**Table 2.** Analysis 1: Sociodemographic comparisons of mail-based vs. E-consent enrollment

Sociodemographic Characteristics	Unadjusted models			Final adjusted model		
	OR	95*%* CI	*p*	AOR	95% CI	*p*
**Age** (*continuous*)	1.02	(1.00, 1.03)	**0.035**	1.02	(1.00, 1.03)	**0.016**
**Education**						
Some college or less	2.46	(1.57, 3.86)	**< 0.001**	2.23	(1.38, 3.58)	**< 0.001**
College degree or more (*referent*)	–	–	**-**	–	–	**-**
**Income**						
Less than $50,000	1.59	(1.10, 2.28)	**0.013**	1.33	(0.91, 1.95)	0.14
$50,000 or more (*referent*)	–	–	–	–	–	–
**Sex**						
Male	0.70	(0.51, .97)	**0.032**	0.75	(0.53, 1.05)	0.09
Female (*referent*)	–	–	–	–	–	–
**Race**						–
White	1.08	(0.77, 1.52)	0.66	–	–	–
non-White (*referent*)	–	–	–	–	–	–
**Ethnicity**						–
Non-Hispanic/Latino	0.68	(0.43, 1.06)	0.09	–	–	–
Hispanic/Latino (*referent*)	–	–	–	–	–	–
**Marital Status**						–
Not married	1.30	(0.92, 1.86)	0.14	–	–	–
Married (*referent*)	–	–	–	–	–	–
**Employment Status**						
Not employed	1.22	(0.88, 1.68)	0.23	–	–	–
Employed (*referent*)	–	–	–	–	–	–
**RUCA**						
Metropolitan	1.00	(0.49, 2.25)	0.99	–	–	–
Micropolitan/small town/rural	–	–	–	–	–	–

RUCA, Rural-Urban commuting area (Range: 1–10); Metropolitan, 1–3; Micropolitan/small town/rural, 4–10

Final model adjusted for age, education, income, and sex.

**Adjusted Model**. In the final adjusted model, older age (*p* = .016) and less education (*p* < .001) significantly predicted likelihood of enrolling via mail (Table 2).

### Analysis 2: Impact of Unconditional Monetary Incentive on Enrollment

**Figure 2.**
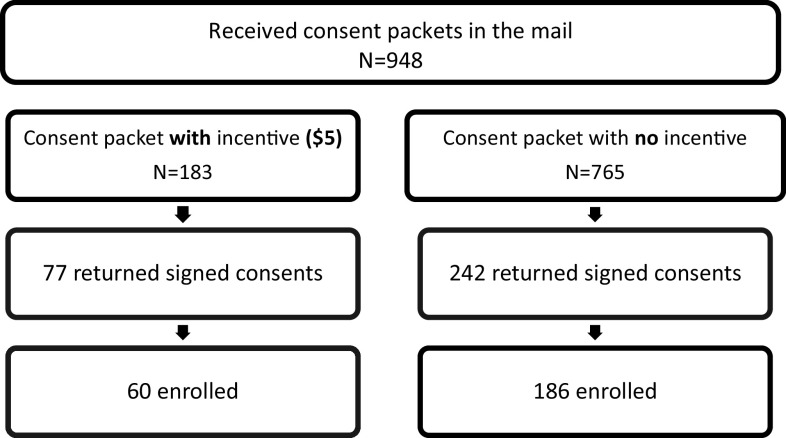
Analysis 2: Flow chart of mail-based study enrollment.

**Sample**. Table 1 also provides descriptive statistics for the demographics of the sample (*N* = 948) who received a mailed consent form, among whom 19.3*%* (*n* = 183) were randomized to the unconditional monetary incentive and 80.7*%* were not (*n* = 765) (Fig. 2).

**Unadjusted model**. Receiving a monetary incentive (vs not) was associated with an increased odds of study enrollment (OR = 1.52; 95*%* CI = 1.07, 2.16, *p* = .019). About one-third of those in the incentive group (*n* = 183; 32.8%), compared to less than one-fourth of those in the non-incentive group (*n* = 765; 24.3%) enrolled in the trial.

**Adjusted model**. Adjusting for sociodemographic covariates of enrollment (i.e., sex, age), monetary incentive (vs. not) remained significantly associated with increased odds of enrolling into the study (AOR = 1.64, 95% CI: 1.14, 2.34, *p* = .007).

### Analysis 3: Implemental Cost-Effectiveness Analysis (ICER)

Our base-case analyses yielded an ICER of approximately $59 additional cost per additional participant enrolled with the $5 incentive (Table 3). This ICER was calculated by dividing the difference in implementation costs by the difference in participants enrolled in the $5 incentive vs. non-incentive groups, or $5000/85 = $58.82. Chi-square test revealed that the 95% CI around the difference in enrollment rates between the groups was 1.05% and 15.95%, equating to a difference of 10 to 159 additional participants enrolled. Therefore, based on our trial results, the ICER could range from an additional $31.45 ($5000 /159) to $500 ($5000/10) per additional participant enrolled.

**Table 3.** Analysis 3: incremental cost-effectiveness ratios (ICER)

Impact on enrollment			Costs	
	Number enrolled*	Additional participants enrolled with incentive	Additional costs with incentive	ICER
No incentive	243	–		
$5 incentive (95% *CI*)	328	85 (10–159)	$5,000 **	$58.82 (31.45–500.00)

ICER, incremental cost-effectiveness ratio.

We calculated 95% “Confidence Intervals” (CIs) around ICER using Chi-Square Difference in Proportion test to set the upper and lower bounds of additional participants enrolled.

* To calculate the ICER in terms of additional cost per additional participant enrolled, the two groups (incentive and non-incentive) must have equal number of subjects. Otherwise, a given group may have more participants enrolled simply because of a larger sample size even if it proportionally enrolls fewer participants. Therefore, we calculated the “number enrolled” by multiplying the trial-derived proportions of participants enrolled in each group by 1,000.

** For the reason described above*, the incremental cost of the incentive group was calculated as $5 per participant * 1,000 as well. Additional costs have no CIs because our trial fixed the incentive payment at $5.00.

## Discussion

Within the context of a larger nationwide remote clinical trial of adult smokers, this study identified sociodemographic characteristics associated with enrolling via mail-based informed consent vs technology-based informed consent (i.e., e-consent). Although enrolling with mailed consent forms was less common than e-consent overall, mail-based enrolling was disproportionally represented by those of older age, less education, lower income, and female sex. After adjusting for covariates, older age and less education remained predictive of enrolling via mail rather than technology-based procedures. This finding is consistent with previous work indicating older adults report lower acceptability of e-consent and less digital literacy [18,19] compared to younger adults [10]. With possible transition to exclusive use of e-consents in remote trials, there is concern that older adults, an age group already largely underrepresented in clinical trials [4], will enroll at lower rates. Further, older age and less education are characteristics associated with lower rates of mobile device ownership and access to home broadband Internet access [6]. Although participants in this study were required to have access to email and/or a mobile device with the capacity to receive SMS text and Internet access (i.e., inclusion criteria in parent trial), those with less education and of older age were likely less familiar with using these technologies. Although e-consent can increase geographical study reach to some populations (e.g., those in rural settings) by diminishing logistical and time constraint barriers to completing consent in-person [4], findings raise concern that e-consent procedures may leave some groups behind. Perhaps, providing mail-based enrollment is one method of offering study participation into remote trials for individuals with technology-based barriers.

Among those receiving mailed consent, a small unconditional monetary incentive ($5 vs no incentive) increased the likelihood of enrollment by 9%. For comparison, Free and colleagues (2010) found that an unconditional €5 (∼$5 USD as of this writing) increased trial enrollment by 4% [20], but Fairhurst and colleagues (2021) found that the same incentive did not increase enrollment [14]. The study presented here is more consistent with the trial conducted by Free *et al* (2010), such that only individuals who were screened eligible and interested in the study were subsequently mailed consent documentation with monetary incentives [20]. In contrast, Fairhurst *et al* (2021) did not screen individuals prior to sending consent packets with rewards [14] which allowed for some individuals who were either ineligible or who did not express *a priori* study interest to receive monetary rewards. Current findings suggest that screening for eligibility and interest prior to sending consent packets could increase enrollment rate, and therefore the efficiency of trial recruitment. Further, the trial by Free *et al* (2010) was similarly among adult smokers [20], while the trial by Fairhurst and colleagues (2021) [14] was conducted within an ongoing yoga intervention for older adults. Therefore, this study, consistent with prior work in a similar population [20], indicates that a small unconditional monetary reward can increase enrollment among adult smokers interested and eligible for a clinical trial.

The incremental cost-effectiveness analysis indicated an additional $59 per additional participant enrolled with the implementation of a $5 incentive. Because all who received the $5 incentive did not necessarily enroll into the study, this additional cost accounts for the “free money” sent to those who did not enroll. In prior trials, additional cost per additional participant enrolled was €1000 (i.e., $1024) [14] and €122 (i.e., $129) [20]. Whether the cost of an unconditional monetary incentive is “worth it” depends upon a study’s priorities, budget, and timeline. This $59 additional cost should be considered within the context of the high overall costs of clinical trials. For example, in a 2018 meta-analysis of costs associated with RCTs, the median cost per participant recruited was $409 (range $41–$6990), and overall costs were between $200,000 and $611.5 million [21]. On the other hand, under-recruitment can lead to insufficient statistical power to achieve study aims. For researchers wanting to both improve age and educational representation of their samples, as well as increase recruitment efficiency, providing the option for mail-based consenting with a $5 unconditional reward might be cost effective.

This study was conducted within a nationwide RCT of adults who smoke cigarettes; therefore, current findings might be less generalizable to other studies or other populations. Additionally, at the time of this study, the local IRB did not require a synchronous phone call with study staff during mail-based enrollment. Dissimilarly, e-consent procedures required scheduling an appointment between study staff and enrollee to allow a synchronous conversation. Therefore, differences in mail-based and e-consent were not solely based on technology but also included the inclusion (or not) of an appointment with study staff. Future studies might consider asking potential participants to report specific reasons for preference (e.g., convenience, lack of technology) of mail-based consenting. Further, eligibility criteria of the parent trial required regular use of email and/or having a mobile device with the capacity to receive SMS text and Internet access. Thus, potential participants needed to have at least some baseline access to technology. The vast majority (93%) of U.S. adults use the Internet [6], so most adults enrolling into clinical trials will have comparable capacity. Regardless, future trials should also assess sociodemographic differences in mail-based vs. e-consent enrollment within the context of trials with no technological inclusion criteria. Further, our sample lacked representation of those living in non-metropolitan areas, which might have contributed to the lack of relationship between rurality and enrollment. Future studies should assess the impact of rurality on mail-based enrollment vs. e-consent within samples including more individuals living in rural areas. Finally, our cost-effectiveness analyses are specific to the magnitude of our reward ($5), which may or may not yield comparable ICER outcomes with different magnitudes.

## Conclusion

E-consents were the dominant, preferred method of recruiting within this remote clinical trial of adult smokers. Mail-based enrollment was less common overall, but disproportionally completed by those older and with less education. Although only a quarter of individuals who received a consent packet in the mail successfully enrolled, a $5 unconditional incentive (vs none) increased the likelihood of enrollment by 9%. As e-consent methods become more common within remote clinical trials, they have the potential to reach many individuals and with greater efficiency, but perhaps at a cost of diminished inclusion across all sociodemographic groups. The provision of an unconditional monetary incentive is possibly a cost-effective mechanism to increase study efficiency for those studies that employ mail-based consenting procedures.
